# Parotid oncocytosis presenting with a spontaneous abscess: a case report

**DOI:** 10.31744/einstein_journal/2026RC2311

**Published:** 2026-09-18

**Authors:** Marcelo Soares Schalch, Rafael de Cicco, Alexandre Bezerra dos Santos, Rafael Pereira de Souza, João Guilherme Ramos Gubert, Tabata Briaunys Milan

**Affiliations:** 1 Department of Head and Neck Surgery Instituto do Câncer Dr. Arnaldo Vieira de Carvalho São Paulo SP Brazil Department of Head and Neck Surgery, Instituto do Câncer Dr. Arnaldo Vieira de Carvalho, São Paulo, SP, Brazil.; 2 Department of Head and Neck Surgery Hospital Santa Paula São Paulo SP Brazil Department of Head and Neck Surgery, Hospital Santa Paula, São Paulo, SP, Brazil.

**Keywords:** Parotid gland, Abscess, Salivary gland neoplasms, Hyperplasia, Oxyphilic cells

## Abstract

Salivary oncocytic lesions are rare entities, representing less than 1% of all salivary gland tumors and predominantly affecting the parotid gland. While oncocytomas are the most common presentation, oncocytic hyperplasia (oncocytosis) is a rarer, multifocal process with virtually no risk of malignant transformation.

**Case Report:**

We report the case of a 69-year-old woman patient who presented with a painful cervical mass and signs of inflammation, initially diagnosed as complicated parotitis. Imaging revealed a heterogeneous mass in the left parotid gland. The patient underwent drainage of a spontaneous abscess associated with the lesion. Histopathological analysis was consistent with diffuse oncocytic hyperplasia (oncocytosis). The patient recovered well after this conservative approach, without the need for total parotidectomy, and remained recurrence-free at the 6-month follow-up.

**Conclusion:**

This case is notable for the unusual presentation of oncocytosis complicated by a spontaneous abscess, an association not previously described in the literature. Accurate diagnosis of this benign condition is crucial for differentiating it from malignant lesions, thereby preventing unnecessary radical surgical procedures and the resulting morbidity.

## INTRODUCTION

Salivary oncocytic lesions are rare entities, representing less than 1% of all salivary gland tumors.^(1)^ These lesions mainly affect the parotid gland^(2)^and include benign oncocytomas, oncocytic carcinomas, and oncocytosis also known as oncocytic hyperplasia.^(3)^

The World Health Organization classifies oncocytic neoplasms into three categories: oncocytoma (62% of cases), a benign, circumscribed tumor that frequently has a central scar; oncocytic hyperplasia or oncocytosis (28.5% of cases), a multifocal process involving diffuse replacement of the parotid gland by oncocytic lobules, with small intervening foci of residual normal serous glands and ducts, and having a less organized and less circumscribed structure than oncocytoma and oncocytic carcinoma (9.5% of cases), which shares histological features with benign oncocytoma but exhibits perineural, intravascular, or lymphatic invasion, frequent mitotic figures, cellular pleomorphism and extensive invasion and destruction of adjacent tissues, making it the rarest of the three categories.^(1,4,5)^

Accurate classification of oncocytic lesions can prevent patients from undergoing unnecessary surgical excision of the affected gland, since oncocytosis has virtually no risk of malignant transformation.^(4)^ However, in addition to being rare, oncocytosis is difficult to diagnose requires assessment using imaging, cytology, histology, and immunohistochemistry. Imaging examinations generally lack sufficient sensitivity or specificity for the definitive diagnosis of oncocytosis. Magnetic resonance imaging (MRI), computed tomography (CT), and even positron emission tomography (PET) are often insufficient to differentiate this disease from malignant tumors. Some lesions may show focal fluorodeoxyglucose (FDG) uptake on PET-CT, potentially resulting in false-positive findings.^(6,7)^Oncocytosis may present as a focal or diffuse lesion of the salivary gland, mainly involving the parotid gland. It shows no sex predilection and is most common between the sixth and eighth decades of life.^(8)^

Oncocytosis a benign course and hyperplastic pathogenesis, unlike oncocytoma, which can be considered a benign salivary gland tumor.^(8)^It is still debated whether oncocytosis represents a true neoplasm proper, an exacerbation of the metaplastic oncocytosis process, or a manifestation of cellular senescence.^(4,9)^

Fine-needle aspiration (FNA) is often insufficient for a precise diagnosis. It has an approximate sensitivity of 30%, and immunohistochemical staining and ultrastructural examination may be required.^(1,10)^Thus, morphology remains essential for definitive diagnosis since there is limited information regarding the immunohistochemical profile of these lesions is limited.^(9)^ Microscopic analysis of oncocytic neoplasms shows a proliferation of uniform polygonal cells with centrally positioned nuclei, and abundant granular eosinophilic cytoplasm, reflecting mitochondrialaccumulation, resulting from a compensatory response to impaired adenosine triphosphate production in oncocytic cells.^(2,11,12)^Histochemical analysis may demonstrate reactivity phosphotungstic acid-hematoxylin.^(9)^

Oncocytosis is characterized byunencapsulated nodules of round oncocytic cells, with granular and eosinophilic cytoplasm, oval nuclei, and prominent nucleoli.^(9)^

Although intratumoral hemorrhage associated with parotid oncocytoma, this is the first reported case of a parotid abscess associated with diffuse oncocytosis.^(13)^The rarity of this condition, combined with the difficulty of establishing a clinicalpathological diagnosis and its association with a spontaneous abscess, makes this case relevant to the medical literature. Its description may assist in the diagnosis of similar cases by establishing oncocytosis as a potential differential diagnosis.

## CASE REPORT

A 69-year-old woman patient was admitted to the hospital with a diagnosis of complicated parotitis in May 2019. She reported local pain and facial edema. Physical examination revealed well defined cervical mass in the left parotid region. The mass was indurated, with no evidence of fluctuation, caused bulging of the left lateral oropharyngeal wall. No cervical lymphadenopathy was detected. Treatment with ceftriaxone and clindamycin was initiated.

On the second day of hospitalization, image-guided aspiration of the collection was performed, and corticosteroids were initiated because of the suspected diagnosis of a parotid tumor complicated by an abscess.

The aspirated material was submitted for cytological analysis. The patient reported significant improvement in pain and edema after aspiration, and physical examination showed a reduction in the size of the mass. Postaspiration CT (Figure 1) demonstrated a decrease in the size of the parotid abscess. However, the parapharyngeal and retroparotid components remained unchanged in size, leading to the decision to surgical drainage.

**Figure 1:**
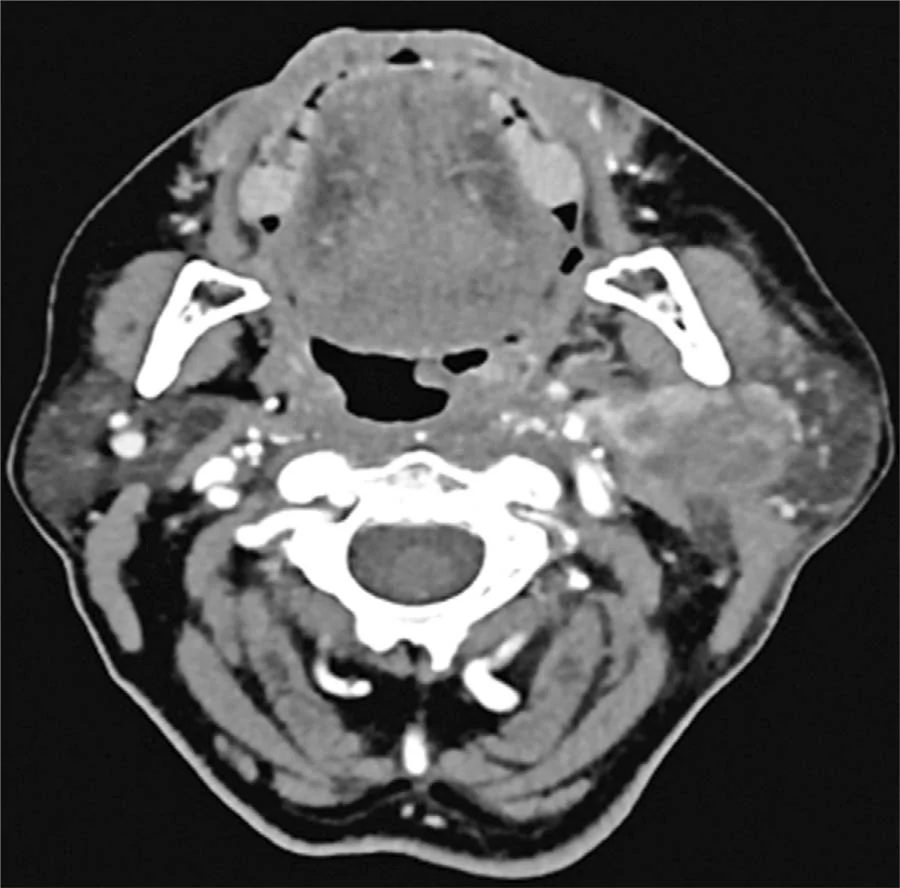
Post-puncture computed tomography of the neck postaspiration computed tomography scan of the neck

This study was approved by the Institutional Ethics Committee of the *Instituto do Câncer Dr. Arnaldo Vieira de Carvalho*, (CAAE: 58015722.3.0000.5471; #5.502.805). Informed consent was obtained from the patient for the publication of this report.

The patient underwent drainage of the pharyngeal abscess and a parotid biopsy on May 20, 2019, followed by placement of a Penrose drain. The surgical procedure was performed using a conventional transcutaneous open approach, providing direct access to the parotid gland. A Penrose drain was positioned within the parotid bed and exteriorized through the cervical skin. There were no intraoperative complications or signs of facial nerve injury. On the second postoperative day, worsening of the oropharyngeal bulging was observed, and a new CT scan revealed a small hematoma; which was managed conservatively with cold compresses. Antibiotic therapy with ciprofloxacin and clindamycin was maintained, and the microbiological cultures were negative.

On the fifth postoperative day, the patient presented significant improvement in symptoms and local edema and was discharged after drain removal, with instructions to continue antibiotic therapy until completion of the prescribed regimen. Table 1 shows the patient’s laboratory findings during hospitalization.

**Table 1:** Laboratory results during hospitalization

Laboratory test results	May 15, 2019	May 17, 2019	May 19, 2019	May 25, 2019
Hemoglobin (g/dL)	16.5	14.8	13.5	15.6
Hematocrit (%)	48.5	42.7	40.2	46.2
Leukocytes (/µL)	10,800	7,300	7,900	9,900
Creatinine (mg/dL)	-	-	0.77	0.92
Amylase (U/L)	34	-	-	-
CRP (mg/dL)	7.6	9.66	1.57	0.98

CRP: c-reactive protein.

Histopathological examination revealed an oncocytic epithelial proliferative lesion extending to the resection margins, with scant residual unremarkable parotid gland tissue, findings consistent with oncocytoma or nodular oncocytic hyperplasia (Figures 2A and 2B).

**Figure 2:**
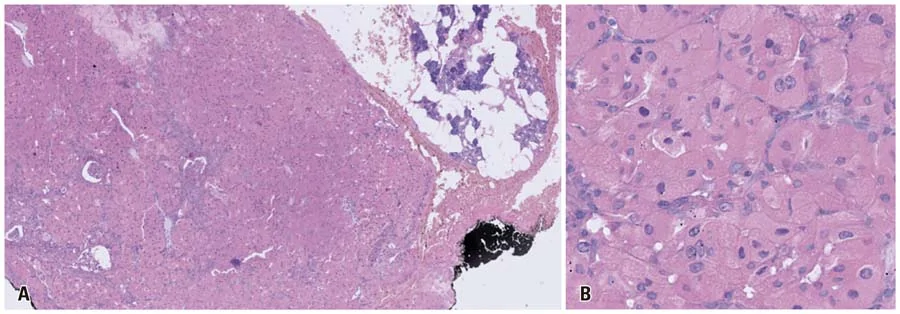
Histopathological features of the parotid biopsy. (A) Low-power view showing an oncocytic epithelial proliferative lesion with scant residual normal parotid gland tissue. (B) High-power view detailing the uniform polygonal cells with centrally positioned nuclei and abundant granular eosinophilic cytoplasm, features characteristic of a oncocytosis lesion (hematoxylin and eosin stain)

The patient remained free of clinical and radiological signs of recurrence, and follow-up MRI showed no recurrent lesions at 6 months of follow-up (Figures 3A and 3B).

**Figure 3:**
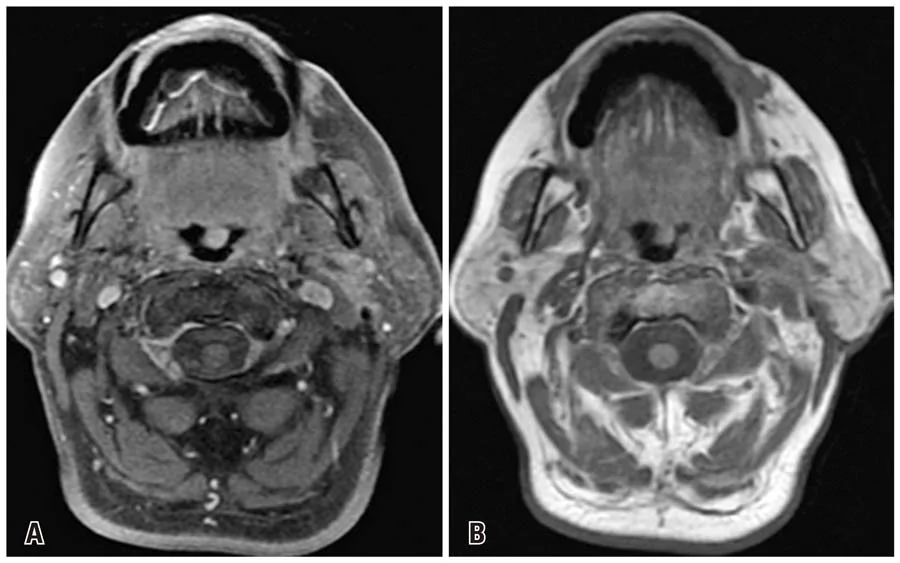
Follow-up magnetic resonance imaging. (A, B) Axial control magnetic resonance imaging scans obtained at the 6-month follow-up, showing postoperative changes in the left parotid bed, with no radiological evidence of lesion recurrence

## DISCUSSION

We present the case of a 69-year-old woman with parotid oncocytosis associated with a local abscess. According to the literature, oncocytosis has no sex predilection, and it is most commonly reported between the sixth and eighth decades of life, consistent with the epidemiological characteristics of this case.^(8)^

Although benign oncocytic lesions are generally slow-growing and painless, local infection has rarely been reported. Hamada et al. described a case of parotid oncocytoma, a true benign neoplasm, in which secondary infection and inflammation led to rapid tumor enlargement and subsequent facial nerve paralysis.^(14)^Our case differs significantly because it involves diffuse oncocytosis which is a hyperplastic process, rather than a circumscribed oncocytoma. While the report by Hamada et al. highlights infection in a neoplastic context, our findings illustrate a unique association between an infectious abscess and nonneoplastic oncocytic hyperplasia, a clinical scenario not previously documented in the literature.

The most distinctive feature of this case was its clinical presentation. Benign parotid tumors generally present as slow-growing, painless, and mobile unilateral masses, although bilateral involvement may occur. The initial clinical presentation in this case was unusual, and the association of oncocytosis with an abscess has not previously been described in the literature. Parotid oncocytosis is a rare condition that is difficult to diagnose.^(6)^

The diagnosis was established by histopathological examination of tissue obtained through an open biopsy, which is considered the gold standard for definitive diagnosis. The favorable clinical course during follow-up further supported this diagnosis. According to Loreti et al.,^(4)^oncocytosis has virtually no risk of malignant transformation, and it remains unclear whether this entity is a true neoplasm or a form of cellular hyperplasia with no malignant potencial.

If an oncocytoma had been diagnosed in the present case, its management and follow-up would have been different, because lesion recurrence might occur, after abscess drainage and biopsy without complete surgical excision, which is the treatment of choice for this neoplasm. In such a cases, complete resection of the lesion is generally indicated, as the recurrence rate is approximately 20% to 30%.^(15)^ Clinical and imaging follow-up with MRI at 1 and 2 years may also be indicated to monitor for recurrence.^(9)^

Oncocytosis, in contrast, is a rare condition with a benign course and no established risk of malignant transformation. The main challenge in its management and follow-up is establishing an accurate diagnosis and differentiation it from other parotid neoplasms, many of which are also benign. A definitive diagnosis is essential because it affects patient treatment and follow-up. In cases of oncocytosis, complete surgical excision may not be necessary, thus avoiding unnecessary surgery its potential complications. A confirmatory diagnosis is particularly important when the patient declines surgery or has substantial surgical risk, because surgery would generally not be indicated for isolated oncocytosis. In the present case, once the diagnosis was established, no additional surgical intervention was considered necessary.

Because to the rarity of this condition and the absence of similar reports, we cannot excluded the possibility that this was a parotid abscess with incidental oncocytosis. Similarly, it cannot be concluded from this single case that oncocytosis is a risk factor for parotid infections. In our literature review, we found no reports of oncocytosis associated with local infection. The rarity oncocytosis and its unusual presentation as a spontaneous abscess in the parotid region make this case relevant to the medical literature.

## CONCLUSION

This case illustrates an unusual presentation of parotid oncocytosis complicated by a spontaneous abscess, an association not previously described in the literature. It reinforces the importance of including oncocytic lesions in the differential diagnosis of parotid masses, even when signs of inflammation signs are present. Histopathological differentiation of oncocytosis from true neoplasms is essential for guideing appropriate management. As demonstrated by the patient’s favorable recovery, a conservative approach that avoids radical parotidectomy may be safe and effective for this benign condition, thereby preventing the morbidity associated with unnecessary extensive surgery.
